# Supplementary material for: Physico-chemical characterization of caesium and strontium using fluorescent intensity of bacteria in a microfluidic platform
Source: R Soc Open Sci. 2019 May 1;6(5):182069. doi: 10.1098/rsos.182069 (PMC6549985; doi:10.1098/rsos.182069)
Supplement: Physico-chemical characterization of Cesium and Strontium using fluorescent intensity of bacteria in a microfluidic platform [file rsos182069supp1.doc]

**Supplementary data**

Physico-chemical characterization of Cesium and Strontium using fluorescent intensity of bacteria in a microfluidic platform

Changhyun Roh ^1,2 †^,Thi T. Nguyen ^3,†^, Jae-Jin Shim ^3^,*and Chankyu Kang ^4^,*

^1^ Decommissioning Technology Research Division, Korea Atomic Energy Research Institute (KAERI), 989-111 Daedukdaero, Yuseong, Daejeon 34057

^2^ Biotechnology Research Division, Advanced Radiation Technology Institute (ARTI), Korea Atomic Energy Research Institute (KAERI), 29 Geumgu-gil, Jeongeup, Jeonbuk 56212

^3^ School of Chemical Engineering, Yeungnam University, 280 Daehak-ro, Gyeonsan, Gyeongbuk 38541

^4^ Office for Government Prime Minister’s Secretariat, Service for promoting safety of people’s lives, 261 Dasom-ro, Sejong 30107


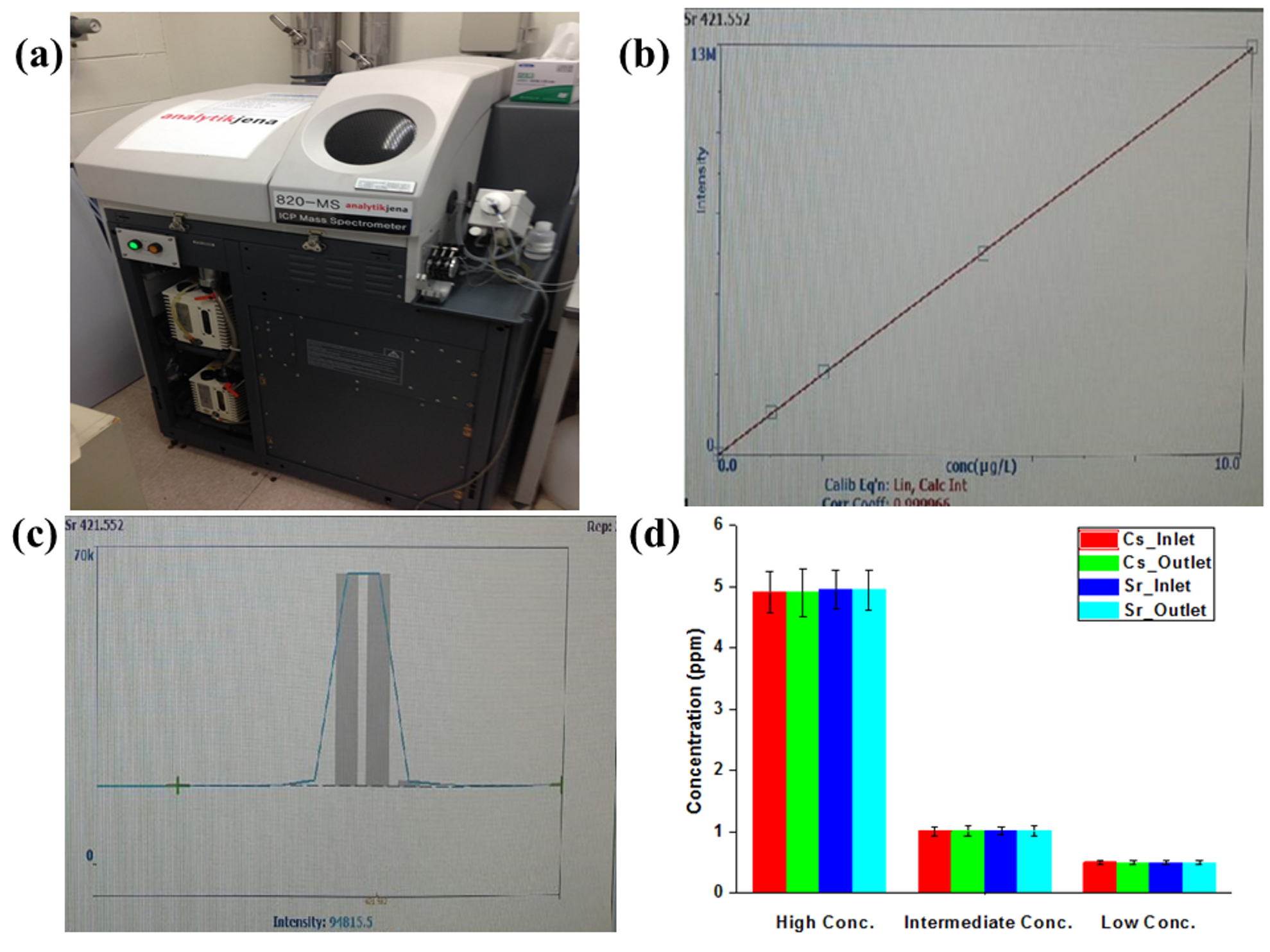


**Figure S1.** (a) ICP/MS for quantitative analysis of CsCl and SrCl_2_ concentrations by ICP/MS, (b) standard curve of SrCl_2_, (c) comparison of SrCl_2_ in the inlet and outlet, and (d) analysis of SrCl_2_.


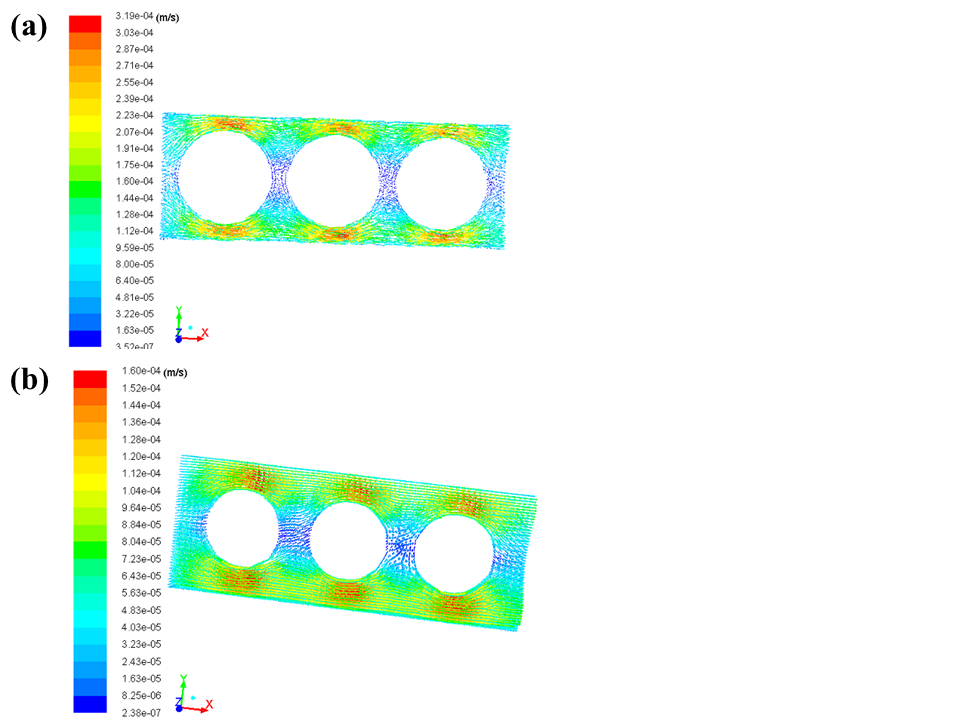


**Figure S2.** CFD simulated flow velocity distribution on the surface of circular microstructures with a diameter (a) 172μm (CM2) and (b) 132μm (CM4).
